# A Novel pyroptosis-related signature for predicting prognosis and evaluating tumor immune microenvironment in ovarian cancer

**DOI:** 10.1186/s13048-023-01275-2

**Published:** 2023-09-20

**Authors:** Jiani Yang, Chao Wang, Yue Zhang, Shanshan Cheng, Yanna Xu, Yu Wang

**Affiliations:** 1 Department of Gynecology, Shanghai First Maternity and Infant Hospital, School of Medicine, Tongji University, Shanghai, China; 2 Shanghai Key Laboratory of Maternal Fetal Medicine, Shanghai Institute of Maternal-Fetal Medicine and Gynecologic Oncology, Shanghai First Maternity and Infant Hospital, School of Medicine, Tongji University, Shanghai, 200092 China; 3 Department of Obstetrics and Gynecology, Renji Hospital, School of Medicine, Shanghai Jiaotong University, Shanghai, China

**Keywords:** Pyroptosis, Ovarian cancer, Tumor immune microenvironment, Prognostic signature

## Abstract

Ovarian cancer (OV) is the most fatal gynecological malignant tumor worldwide, with high recurrence rates and great heterogeneity. Pyroptosis is a newly-acknowledged inflammatory form of cell death with an essential role in cancer progression, though studies focusing on prognostic patterns of pyroptosis in OV are still lacking. Our research filtered 106 potential pyroptosis-related genes (PRGs) among the 6406 differentially expressed genes (DEGs) between the 376 TCGA-OV samples and 180 normal controls. Through the LASSO-Cox analysis, the 6-gene prognostic signature, namely CITED2, EXOC6B, MIA2, NRAS, SETBP1, and TRPV46, was finally distinguished. Then, the K-M survival analysis and time-dependent ROC curves demonstrated the promising prognostic value of the 6-gene signature (p-value < 0.0001). Furthermore, based on the signature and corresponding clinical features, we constructed and validated a nomogram model for 1-year, 2-year, and 3-year OV survival, with reliable prognostic values in TCGA-OV (p-value < 0.001) and ICGC-OV cohort (p-value = 0.040). Pathway analysis enriched several critical pathways in cancer, refer to the pyroptosis-related signature, while the m6A analysis indicated greater m6A level in high-risk group. We assessed tumor immune microenvironment through the CIBERSORT algorithm, which demonstrated the upregulation of M1 Macrophages and activated DCs and high expression of key immune checkpoint molecules (CTLA4, PDCD1LG2, and HAVCR2) in high-risk group. Interestingly, the high-risk group exhibited poor sensitivity towards immunotherapy and better sensitivity towards chemotherapies, including Vinblastine, Docetaxel, and Sorafenib. Briefly, the pyroptosis-related signature was a promising tool to predict prognosis and evaluate immune responses, in order to assist decision-making for OV patients in the realm of precision medicine.

**Supplementary Information:**

The online version contains supplementary material available at 10.1186/s13048-023-01275-2.

## Introduction

Ovarian cancer (OV) is the second most common gynecological disease and the most fatal gynecological malignant tumor worldwide, thus seriously threatening women’s safety and health [1]. As reported, there were 19,880 new cases and 12,810 deaths related to OV, estimated for 2022 in the United States [2]. Owing to the lack of specific early symptoms, over 70% OV cases were first diagnosed at late period, which led to a poor 5-year overall survival rate of 35% [3, 4]. After the initial therapy of surgery combined with platinum-based chemotherapy, approximately 80% OV patients finally suffer recurrence and progression [5]. Accordingly, to improve survival, identifying a promising prognostic signature is of great urgency.

During the past decades, cell death, one of the most fundamental issues for life sciences, has been defined as a hallmark of cancer [6]. Recently, increasing researches have been focused on pyroptosis, a newly-acknowledged inflammatory form of cell death [7]. Pyroptosis was usually caused by certain inflammasomes, which could lead to the cleavage of Gasdermin D (GSDMD) and maturation of pro-inflammatory cytokines, such as interleukin-18 (IL-18) and interleukin-1β (IL-1β) [8]. With the deepening of studies, the essential role of pyroptosis has been proved in various aspects, including tumor origin, tumor progression, and therapy-resistance, etc. [9]. As for OV and pyroptosis, Berkel and colleagues pointed out that the expression of GSDMD and GSDMC was up-regulated, whereas GSDME was downregulated in OV tissue, which was associated with poor prognosis [10]. In this regard, it is of great importance to explore the underlying mechanisms of pyroptosis-related genes (PRGs) in the process of OV progression, which has guiding significance in the treatment and prevention of cancer [11].

Recently, immunotherapy has become a hotspot in OV studies, though the effective rate for immunotherapy in OV is still limited [12, 13]. Up till now, emerging evidence has suggested the crosstalk between tumor immune microenvironment and pyroptosis [14]. Most researches focused on only one or two pyroptosis-related genes (PRGs) and several cell types in the microenvironment, however, the tumor progression process is characterized by numerous genes and cell types interacting in a high-coordinated manner, which haven’t been fully understood yet [15, 16]. Hence, the in-depth mechanisms of pyroptosis along with the tumor immune microenvironment in OV progression could be instrumental in developing efficacious immunotherapy to overcome drug resistance [17].

Accordingly, in our research, we comprehensively evaluated the importance of PRGs in OV, and filtered 6 PRGs to build a prognostic signature. Moreover, we assessed the difference of methylation N6 adenosine (m6A) level, tumor immune microenvironment, and sensitivity towards chemotherapy/immunotherapy between risk groups classified via the pyroptosis-related signature.

## Methods

### Data collection

**Fig. 1:**
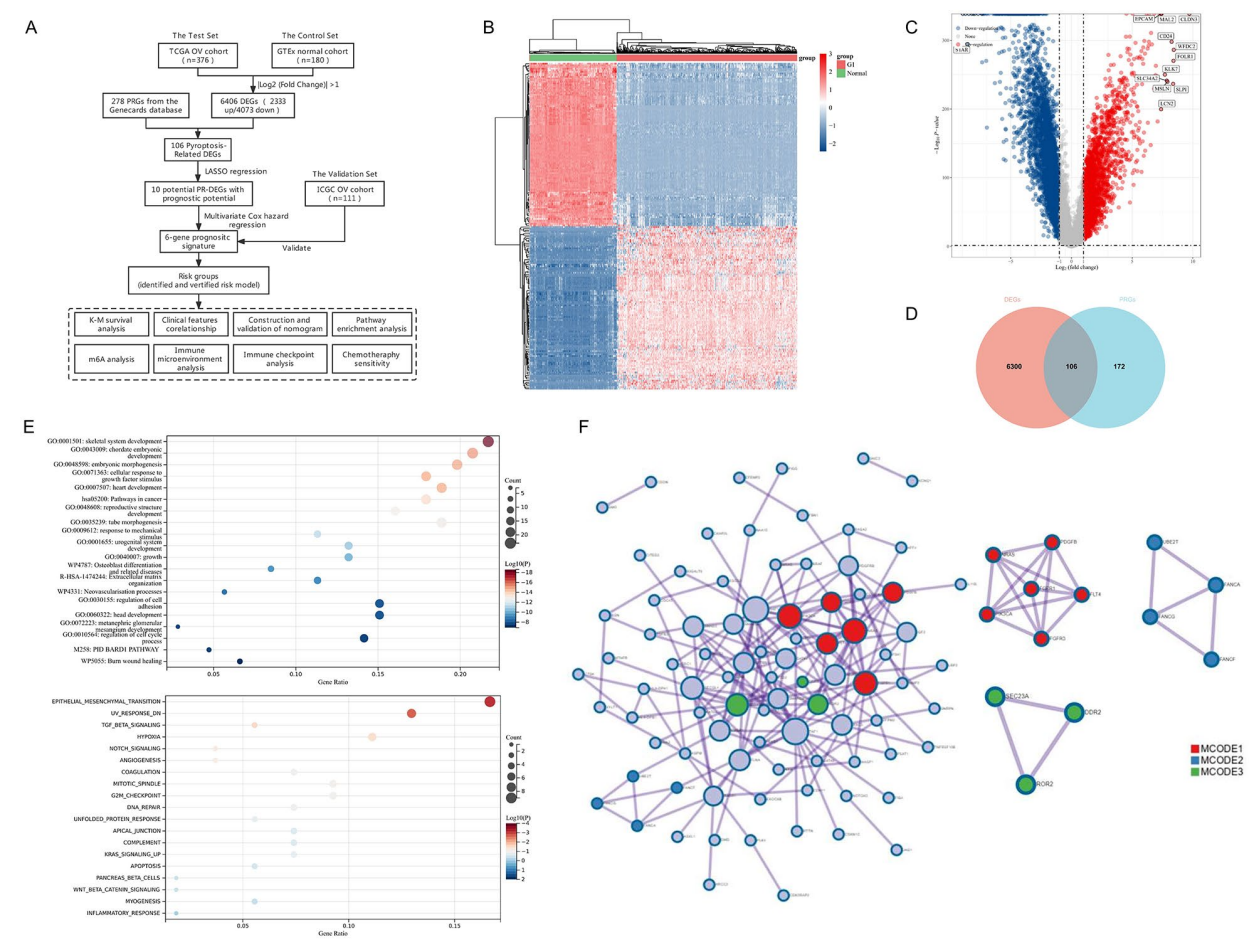
Identification of differentially expressed pyroptosis-related genes (DE-PRGs) in ovarian cancer (OV). (**A**) The flowchart of the research. (**B**) The heatmap of differential gene expression, among which the top 50 up-regulated and the top 50 down-regulated genes were listed. Different colors represent the different trend of gene expression between normal tissues and OV tissues. (**C**) The volcano plot showed the differentially expressed genes (DEGs) between normal tissues and OV tissues. The up-regulated and down-regulated DEGs were respectively highlighted in red and blue. (**D**) The Venn plot of the DE-PRGs. (**E**) The Gene Ontology (GO) and Kyoto Encyclopedia of Genes and Genomes (KEGG) pathway enrichment analysis of the 106 DE-PRGs (up). The hallmark pathway from mSigDB enrichment analysis of the 106 DE-PRGs (bottom). Here, the top 20 clusters were shown, while the size of the circles represents gene ratio and the color scale represents the p-value. (**F**) The protein-protein interaction (PPI) network plot of the 106 DE-PRGs (left), among which 13 hub genes with significant associations were defined (right)

Figure 1 A showed the workflow of the research. The gene expression profiles of OV patients were obtained from The Cancer Genome Atlas database (TCGA, https://portal.gdc.com) as the training cohort (n = 376) and from the International Cancer Genome Consortium database (ICGC, https://dcc.icgc.org) as the validation cohort (n = 111). The corresponding clinical features of OV patients were also publicly available from the TCGA and ICGC datasets, which were summarized in Supplement Table 1. All patients involved underwent standard operation aimed to achieve optimal tumor debulking followed by platinum-based chemotherapy. We also extracted the transcriptome profiles of normal tissues as controls (n = 180), through the Genotype-Tissue Expression database (GTEx, https://gtexportal.org). Based on the “limma” package of the R software, the RNA-sequencing raw data was normalized for further analysis. The batch effects caused by non-biotechnological bias among two different datasets (TCGA and GTEx) were corrected through the “Combat” algorithm using the “SVA” package of the R software [18].

### Filtration of pyroptosis-related genes

From the Genecards database (https://www.genecards.org), we identified 278 ferroptosis-related mRNAs with Relevance Score ≥ 2. Then, we filtered the differentially-expressed genes (DEGs) between normal and OV tissues (adjusted p-value < 0.05; | Log_2_ (Fold Change) | >1). According to the Venn diagram, we identified differentially expressed pyroptosis-related genes (DE-PRGs). To identify the prognosis value of the identified genes, the Kaplan–Meier (K-M) method was used to graph survival curves and the p-value was assessed through the Log-rank test.

### Construction and validation for prognostic signature

To filter prognostic PRGs for signature construction, the least absolute shrinkage and selection operator (LASSO) - COX Regression algorithm was conducted with 10-fold cross-validation, through the “glmnet” package of the R software. We also performed the Time-dependent receiver operating characteristic curve (ROC) analysis of 1-year, 3-year, and 5-year survival rate through the “timeROC” package of the R software. For survival analysis, we stratified OV patients into two risk groups, according to the medium cut-off value of risk-score. Next, the Kaplan–Meier (K-M) analysis was conducted to assess prognostic value of the signature.

To further select independent risk factors for the nomogram, we carried out univariate and multivariate Cox Hazard Regression analyses, which were visualized through the forest diagram using the “forestplot” package of the R software. According to the selected risk factors, we then constructed a nomogram for 1-year, 3-year, and 5-year OS prediction through the “rms” package of the R software.

### Functional enrichment analysis and tumor immune microenvironment analysis

Furthermore, we analyzed the functional enrichment of gene expression profiles, in order to assess the underlying functions of the potential genes. The Kyoto Encyclopedia of Genes and Genomes (KEGG) and Gene Ontology (GO) functional enrichment analyses, including molecular function (GO-MF), biological pathways (GO-BP), and cellular components (GO-CC) were analyzed using the “ClusterProfiler” package of the R software.

To evaluate immune microenvironment of tumor tissue, we analyzed the abundance proportion of 22 typical tumor-infiltrating immune cells, based on the CIBERSORT algorithm at the CIBERSORTx (https://cibersortx.stanford.edu/) website [19] and the Estimation of STromal and Immune cells in MAlignant Tumor tissues using Expression data (ESTIMATE, https://bioinformatics.mdanderson.org/estimate/). Moreover, the correlations between the 22 immune cells signature risk score were calculated through the Spearman’s test.

### Assessment of patient response toward immunotherapy and chemotherapy

To identify effective immunotherapy for OV patients, we conducted the Pearson’s test to evaluate the relationship between the pyroptosis-related signature and expression of immune checkpoint genes, including CD274, CTLA4, HAVCR2, LAG3, PDCD1, PDCD1LG2, SIGLEC15, and TIGIT. In addition, we predicted the Potential immune checkpoint blockade (ICB) response for individuals, based on the TIDE website (http://tide.dfci.harvard.edu/).

Moreover, we analyzed the half-maximal inhibitory concentration values (IC50) through the ridge regression, in order to evaluate patient response to chemotherapy. The chemotherapy-related data was obtained from the Genomics of Drug Sensitivity in Cancer database (GDSC, https://www.cancerrxgene.org/), the largest public pharmacogenomics dataset. The prediction for IC50 was performed through the “pRRophetic” package of the R software.

### Statistical source

The differences between groups were compared through the Chi-squared test for categorical variables and the Wilcoxon test for continuous variables. The Spearman correlation analysis was used to analyze correlations between different variables, and the “ggstatsplot” package of the R software was used to graph the multi-gene correlation heatmap. P-value was adjusted using the BH method, while the p-value (two-tailed) < 0.05 was considered statistically significant. Statistical analyses were performed using the R software (version 4.0.3).

## Results

### Identification of pyroptosis-related differentially expressed genes in OV

Firstly, we downloaded the transcriptome data and corresponding clinical features of OV patients (n = 376) from the TCGA-OV database (https://portal.gdc.com). The transcriptome data from normal tissues (n = 180) was also obtained from the GTEx database (https://gtexportal.org) as controls. As shown in Fig. 1B and C, we identified 6406 DEGs, among which 4073 genes were down-regulated, and 2333 genes were up-regulated in OV tissues compared with the normal tissues. Then, we obtained 278 PRGs (Relevance Score ≥ 2) from the Genecards database (https://www.genecards.org). According to the Venn diagram, 106 PRGs were differentially expressed between normal tissues and OV tissues (Fig. 1D). Then, we conducted a pathway enrichment analysis of the 106 DE-PRGs through the Metascape website (https://metascape.org) [20]. Among the 106 DE-PRGs, 38 genes were up-regulated in the OV cancer tissues compared with normal, while 68 genes were down-regulated in the OV tissues. In Fig. 1E, the top 20 most significant GO and KEGG pathways were listed, which were mainly enriched in pathways in cancer, cellular response to growth factor stimulus, regulation of cell cycle process, etc. Based on the hallmark pathway analysis from the Molecular Signatures Database (mSigDB), the DE-PRGs mainly enriched in pathways including the Epithelial-mesenchymal transition (EMT), TGFβ signal, and hypoxia, which indicated that these pathways might be related to the pyroptosis pattern. In order to provide screens for protein interactions, we conducted a protein-protein interaction (PPI) network for the 106 DE-FRGs, based on the Search Tool for the Retrieval of Interacting Genes (STRING, https://string-db.org) (Fig. 1F) [21]. Among 106 DE-FRGs, 13 hub genes with significant associations were defined.

### Establishment and estimation of the prognostic signature based on pyroptosis-related genes

**Fig. 2:**
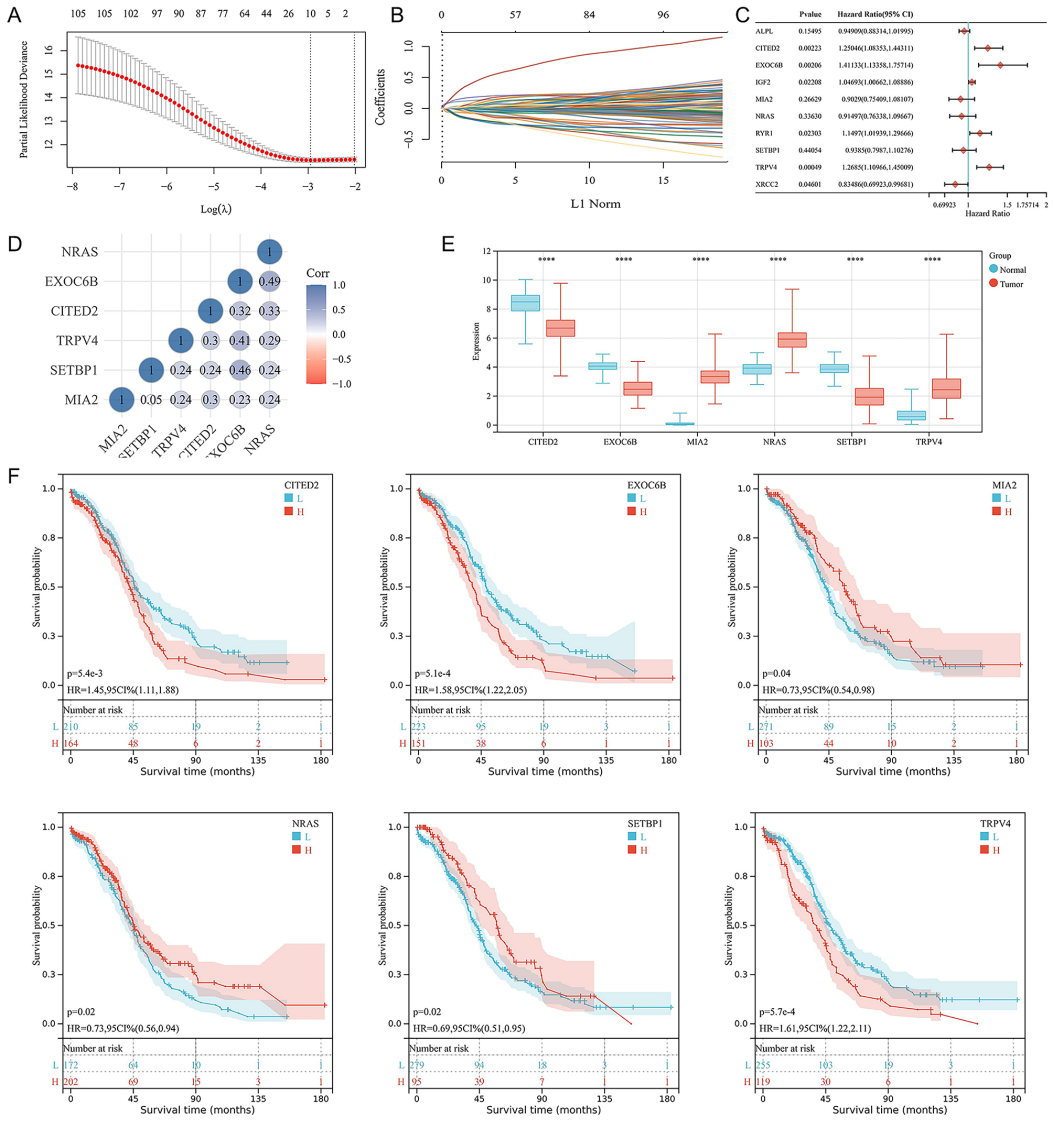
Construction of an ovarian cancer (OV) prognostic signature based on the pyroptosis-related genes (PRGs). (**A**) The λ selection plot of the 10-fold cross-validation for the LASSO tuning parameter selection. (**B**) The LASSO-Cox analysis for the optimal prognostic PRGs, including MIA2, XRCC2, NRAS, ALPL, TRPV4, RYR1, EXOC6B, SETBP1, CITED2, and IGF2. (**C**) The forest plot represented the prognostic ability of the ten optimal PRLs, which were analyzed through the Cox Regression algorithm. (**D**) The heatmap for the relationship among the six prognostic PRGs, namely CITED2, EXOC6B, MIA2, NRAS, SETBP1, and TRPV4. The color scale represented different correlation coefficients (red for negative relationship and blue for positive relationship). (**E**) The expression distribution of the six prognostic PRGs in normal tissues and OV tissues. (**F**) The Kaplan-Meier (K-M) survival curves of the six prognostic PRLs.

Through the LASSO regression analysis, we filtered ten potential prognostic genes (including MIA2, XRCC2, NRAS, ALPL, TRPV4, RYR1, EXOC6B, SETBP1, CITED2, and IGF2) from the 106 DE-FRG (Fig. 2A and B). To enhance model explicability, the multivariate Cox Regression analysis was conducted to distinguish prognostic genes for the signature, namely CITED2, EXOC6B, MIA2, NRAS, SETBP1, and TRPV4 (Fig. 2C). The relationships among these selected DE-PRGs were shown in Fig. 2D, while the expression distribution of the six prognostic PRGs in normal tissues and OV tissues was also presented (Fig. 2E). Ultimately, we constructed the pyroptosis-related 6-gene prognostic signature model as follows: risk-score = (0.2726)*CITED2 + (0.4932)*EXOC6B + (-0.29)*MIA2 + (-0.3169)*NRAS + (-0.2583)*SETBP1 + (0.2071)*TRPV4. As shown in the Kaplan-Meier (K-M) survival curves (Fig. 2F), OV patients with high expression of MIA2, NRAS, and SETBP1 had better OS, while those with high expression of CITED2, EXOC6B, and TRPV4 suffered worse OS. The overview for the functions in OV of the six DE-FRGs with prognostic values was listed (Supplement Table 2) [22–29]. We have also checked the expression dimension of two merged expression dataset using the PCA analysis, which have been shown in Supplement Fig. 1

**Fig. 3:**
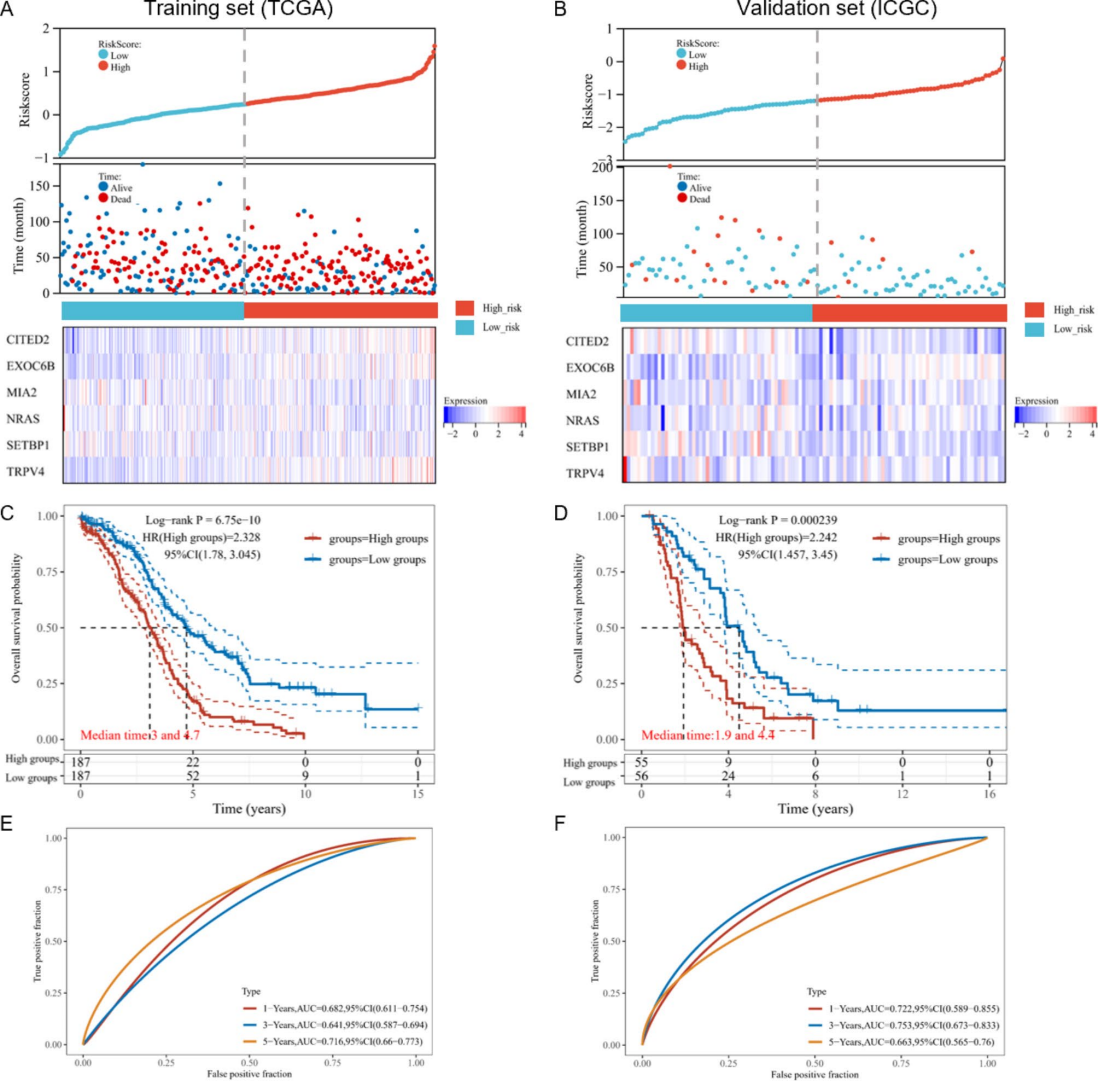
Estimation and validation of the prognostic signature based on the six pyroptosis-related genes (PRGs). The distribution of the risk score, survival time (months), and survival status of ovarian cancer (OV) patients in the TCGA training set (**A**) and the ICGC validation set (**B**). The scatter diagrams represented the risk score of different OV patients, refer to corresponding survival time and survival status (top and middle). The heatmaps (bottom) showed gene expression of the 6-gene signature between low-risk and high-risk groups. The Kaplan-Meier (K-M) curves for overall survival (OS), classified into the low-risk and high-risk groups of the TCGA training set (**C**) and the ICGC validation set (**D**). The ROC analysis of the TCGA training set (**E**) and the ICGC validation set (**F**) of OS prediction by the 6-gene signature based on PRGs.

Through the above formula, we calculated the risk-score of each OV patients, including the training set (TCGA-OV cohort; n = 374) and the validation set (ICGC-OV cohort; n = 111), which were followed-up for 41.76 ± 31.78 months and 41.63 ± 31.16 months, respectively. Then, we divided patients into two groups: low-risk and high-risk, according to the median cut-off value. Figure 3 A and 3B (top and middle) showed the risk-scores of OV patients in both training and validation sets, refer to corresponding survival time and status. We also listed the expression profiles of the 6-gene signature in low-risk and high-risk groups. In both training and validation sets, MIA2 and SETBP1 were highly expressed in the low-risk group, while EXOC6B was mainly expressed in the high-risk group. The K-M curves demonstrated that patients in the low-risk group had better 1-year, 3-year, and 5-year OS in the training cohort (p-value < 0.0001) and validation cohort (p-value = 0.0002) (Fig. 3C and D). Through the time-dependent ROC analysis, we indicated that the 6-gene signature had promising prognostic values for 1-year, 3-year, and 5-year OS prediction (Fig. 3E F).

### Construction and validation of the pyroptosis-related 6-gene‑based nomogram

We analyzed the relationship between the pyroptosis-related 6-gene signature and clinical features, including age (Supplement Fig. 2A), race (Supplement Fig. 2B), grade (Supplement Fig. 2C), and International Federation of Gynecology and Obstetrics (FIGO) stage (Supplement Fig. 2D). The results showed that elder patients (age ≥ 60 years) are more likely to have higher risk-score, while race, grade, and FIGO stage had no significant relationship with the signature (p-value ≥ 0.05). The Sankey plot visualized the distribution of every OV patient, based on the risk groups stratified by the 6-gene signature and corresponding clinical features (Supplement Fig. 2E).

**Fig. 4:**
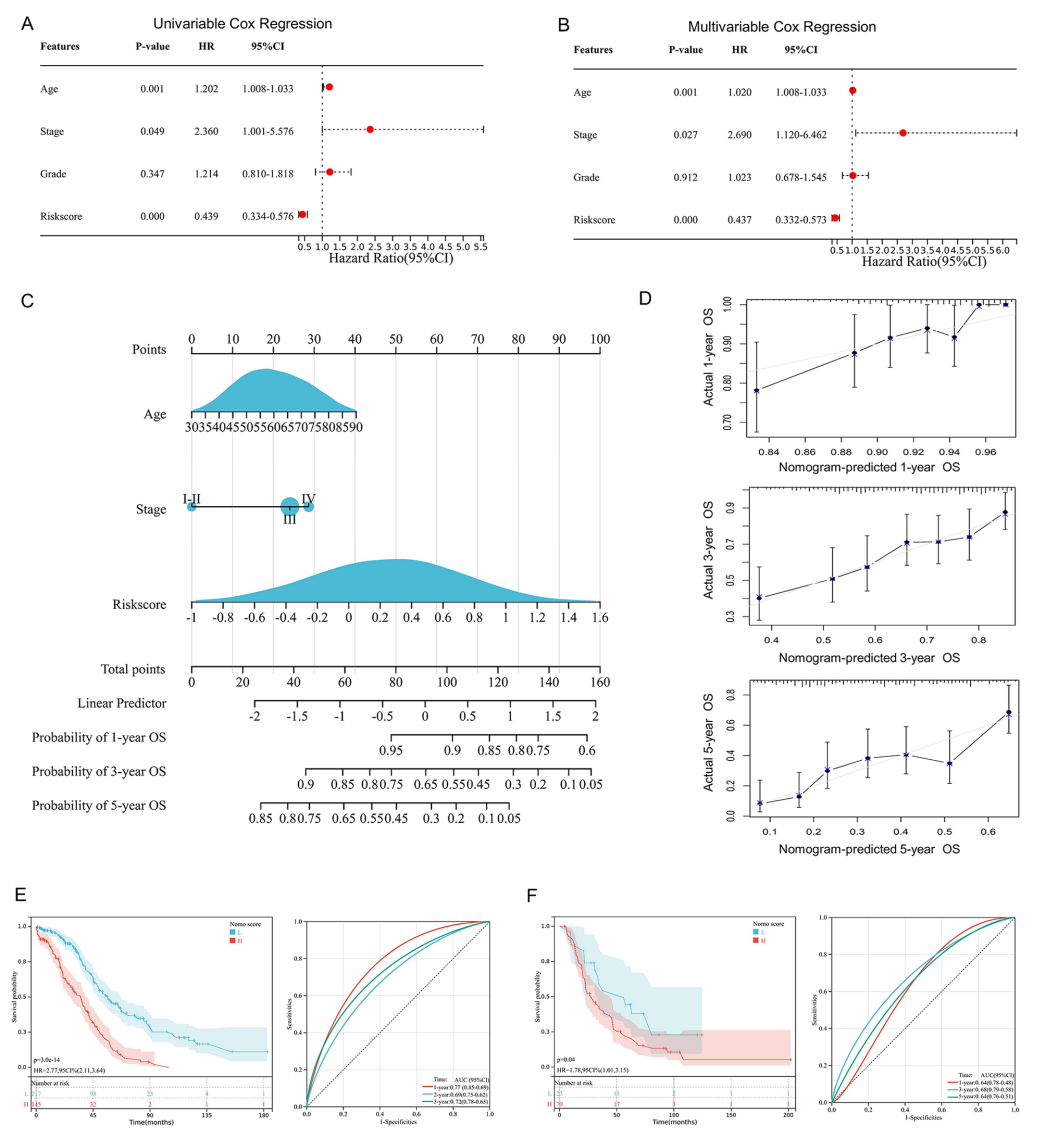
Construction and validation of the pyroptosis-related 6-gene‑based nomogram. The forest plots for univariate (**A**) and multivariate (**B**) Cox Hazard Regression analysis of overall survival (OS), based on the 6-gene signature and clinical characteristics, including age, pathological grade, and clinical FIGO stage. (**C**) The prognostic nomogram model for 1-year, 3-year, and 5-year OS of ovarian cancer (OV) patients, based on the 6-gene risk score and clinical indicators selected by the Cox Regression analysis. (**D**) The calibration diagrams of the prognostic nomogram for predicting 1-year, 3-year, and 5-year OS (top, middle, and bottom) among OV patients. (**E**) The Kaplan-Meier (K-M) curves (left) and Receiver Operating Characteristic (ROC) curves (right) for patients in the TCGA-OV training cohort, classified by the prognostic nomogram score. (**F**) The K-M curves (left) and ROC curves (right) for patients in the ICGC-OV validation cohort, related to the nomogram score

In order to find out prognostic indicators for OV patients, we implied the univariable and multivariable Cox Hazard Regression analyses, which confirmed that age (p-value = 0.001), FIGO clinical stage (p-value = 0.027), and risk-score (p-value < 0.001) were independent prognostic indicators for OS (Fig. 4A and B). Thus, we constructed a prognostic nomogram model for 1-year, 3-year, and 5-year OS probability for OV patients, based on the integration of age, FIGO clinical stage, and 6-gene risk-score, (Fig. 4C). The results indicated that the nomogram had promising C-index of 0.6663 (95%CI 0.6278–0.7048) and 0.6037 (95%CI 0. 5675-0.7045) in the TCGA training cohort and the ICGC validation cohort, respectively. The calibration curves of nomogram model showed great consistency between the predicted and observed 1-year, 3-year, and 5-year OS (Fig. 4D; up, middle, and bottom, respectively). Then, we calculated the prognostic nomogram score for OV patients. Based on the median cut-off value, we stratified patients into two nomogram groups. Furthermore, the K-M survival curves demonstrated that OV patients with low nomogram scores had better OS in both the training cohort (p-value < 0.001, Fig. 4E, left) and the validation cohort (p-value = 0.040, Fig. 4F, left). The ROC curves analysis demonstrated that the nomogram scores had reliable predictive value for OV prognosis in both the training cohort (Fig. 4E, right) and the validation cohort (Fig. 4F, right), with the Area Under Curve (AUC) value of 0.77 and 0.64, respectively. Accordingly, the nomogram model based on the pyroptosis-related 6-gene signature had promising prognostic value for OV patients.

### Pathway enrichment analysis for the pyroptosis-related 6-gene signature

**Fig. 5:**
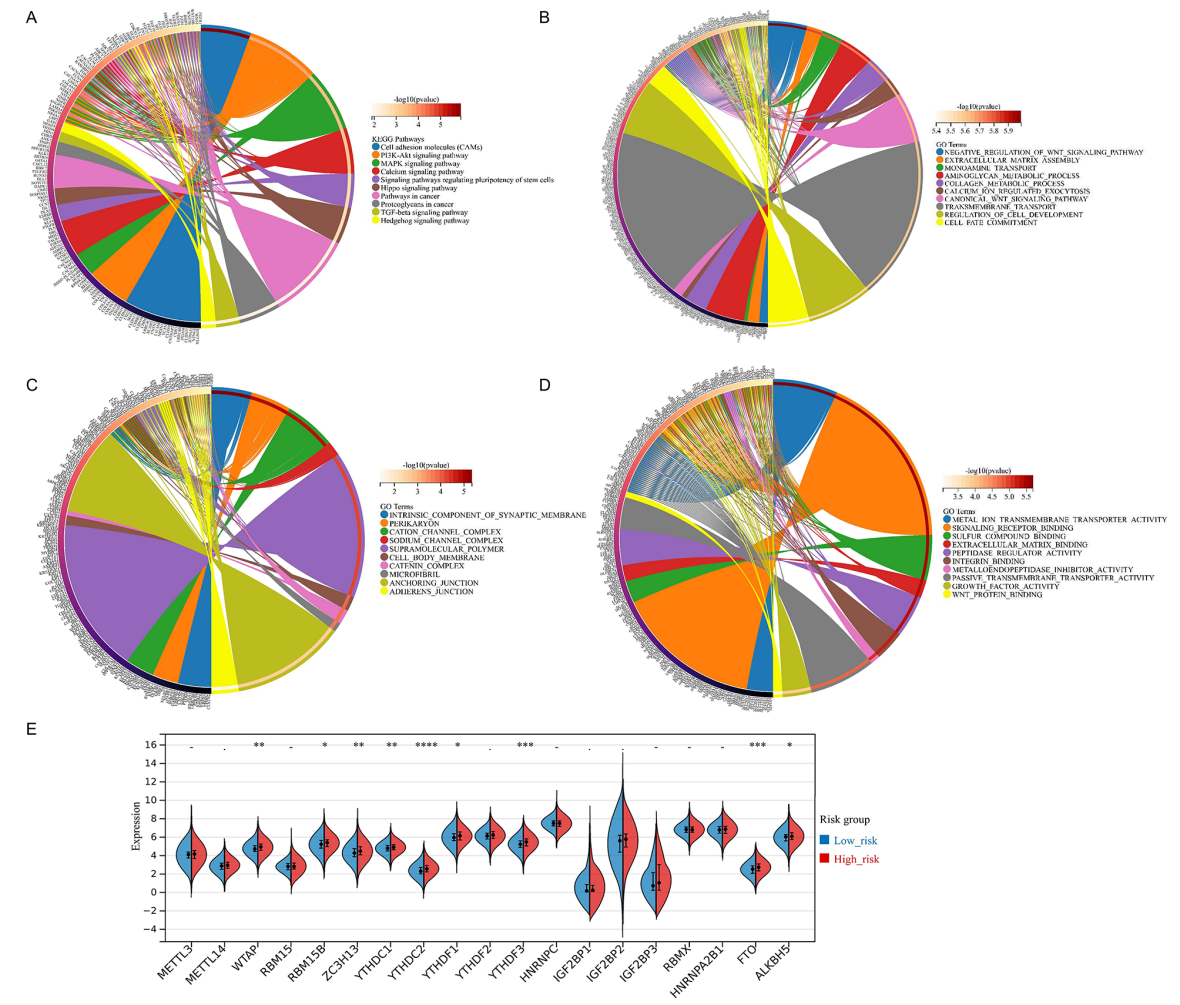
Pathway enrichment analysis and immunity analysis for the pyroptosis-related 6-gene signature. (**A**) The Kyoto Encyclopedia of Genes and Genomes (KEGG) pathway enrichment analysis for potential genes. (**B-D**) The Gene Ontology (GO) pathway enrichment analysis for potential genes in terms of the biological process (BP), the cellular component (CC), and the molecular function (MF). The size of circles indicated gene numbers, while the color scale represented -log10(P-value). (**E**) The violin diagrams represented the expression distribution of the 19 typical N6-methyladenosine (m6A)-associated genes, between low-risk and high-risk groups. *P-value < 0.05; **P-value < 0.01; ***P-value < 0.001; ****P-value < 0.0001

Stepwise, we conducted both KEGG and GO pathway enrichment analysis among OV patients stratified via the pyroptosis-related 6-gene signature. The KEGG pathway analysis enriched several critical pathways in cancer, such as the PI3K-Akt signaling pathway, Hedgehog signaling pathway, MAPK signaling pathway, and others (Fig. 5A). The GO biological process (GO-BP) pathway analysis was significantly enriched in regulation of the WNT signaling pathway, transmembrane transport, and cell fate commitment, etc. (Fig. 5B). The GO cellular component (GO-CC) pathway enrichment analysis identified intrinsic components of synaptic membrane, supramolecular polymer, anchoring junction, and others (Fig. 5C). The GO molecular function (GO-MF) analysis was significantly enriched in pathways including signaling receptor binding, WNT protein binding, and passive transmembrane transporter activity, etc. (Fig. 5D).

Recently, emerging studies implied the critical role of m6A, a common type of RNA modification, in OV progression [30]. Hence, we identified 19 typical m6A-associated genes (including ALKBH5, FTO, HNRNPA2B1, HNRNPC, IGF2BP1, IGF2BP2, IGF2BP3, METTL3, METTL14, RBMX, RBM15, RBM15B, WTAP, YTHDC1, YTHDC2, YTHDF1, YTHDF2, YTHDF3, and ZC3H13) from a study, which focused on molecular characterization of m6A modulators among 33 various cancer types in the TCGA pan-cancer cohort, including OV [31]. Interestingly, 9 out of the 19 m6A-related genes, including WTAP, RBM15B, ZC3H13, YTHDC1, YTHDC2, YTHDF1, YTHDF3, FTO, and ALKBH5 were highly expressed in high-risk group (p-value < 0.05), compared with low-risk group (Fig. 5E).

### Immunity analysis for tumor immune microenvironment related to the 6-gene signature

**Fig. 6:**
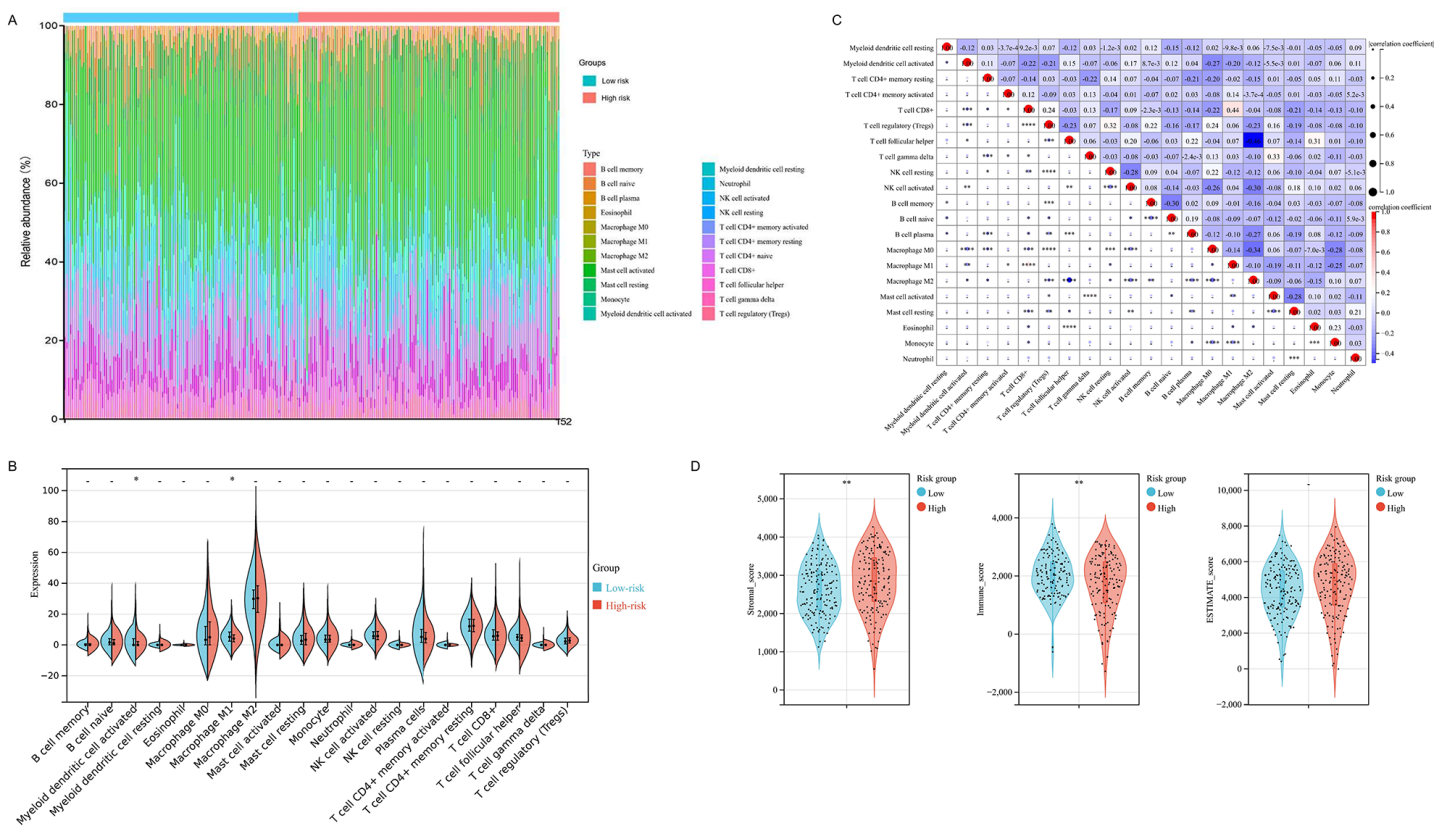
The tumor immune landscape related to the 6-gene signature. (**A**) The Boxplots showed the composition of 22 immune cells infiltrating in OV samples, which were analyzed through the CIBERSORT algorithm. OV patients were classified into low-risk and high-risk groups by the 6-gene signature. (**B**) The Violin diagrams indicated the difference in the 22 immune cells infiltration between low-risk and high-risk groups. (**C**) The heatmaps showed the proportions and relationships of the 22 immune cells among OV patients. (**D**) Based on the ESTIMATE algorithm, the stromal score, immune score, and ESTIMATE score, which infers the presence of stroma, infiltration of immune cells, and tumor purity, were compared among two risk groups. *p-value < 0.05; **p-value < 0.01; ***p-value < 0.001; ****p-value < 0.0001

Growing evidence suggested that tumor immune microenvironment could contributed to OV progression through the crosstalk between proximal immune cells and tumor cells [32]. Therefore, in order to determine the association between the pyroptosis-related 6-gene signature and tumor immune microenvironment, we conducted the CIBERSORT algorithm to evaluate immune infiltration landscape among OV patients, which were stratified by the pyroptosis-related signature. We summarized the composition of 22 immune cells infiltrating in OV samples, from both the low-risk and high-risk groups (Fig. 6A). According to the CIBERSORT analysis, 2 out of the 22 immune cells, including M1 Macrophages and activated Myeloid Dendritic Cells (DCs) were significantly up-regulated in the low-risk group compared to the high-risk group (Fig. 6B).Except for the intense correlation between resting and corresponding activated cells, Follicular Helper T cells and M2 Macrophage had the strongest negative relationship (correlation coefficient 0.46; p-value < 0.0001), while CD8 + T cells and Macrophage M1 had the strongest positive relationship (correlation coefficient 0.44; p-value < 0.0001) (Fig. 6C). Based on the ESTIMATE algorithm, we found that both the stromal score was significantly higher in the high-risk group, while the immune score was higher in low-risk group. However, there was no difference of the ESTIMATE score, which infers tumor purity, among two risk groups (p-value > 0.05, Fig. 6D).

### Assessment of patient response toward immunotherapy and chemotherapy

**Fig. 7:**
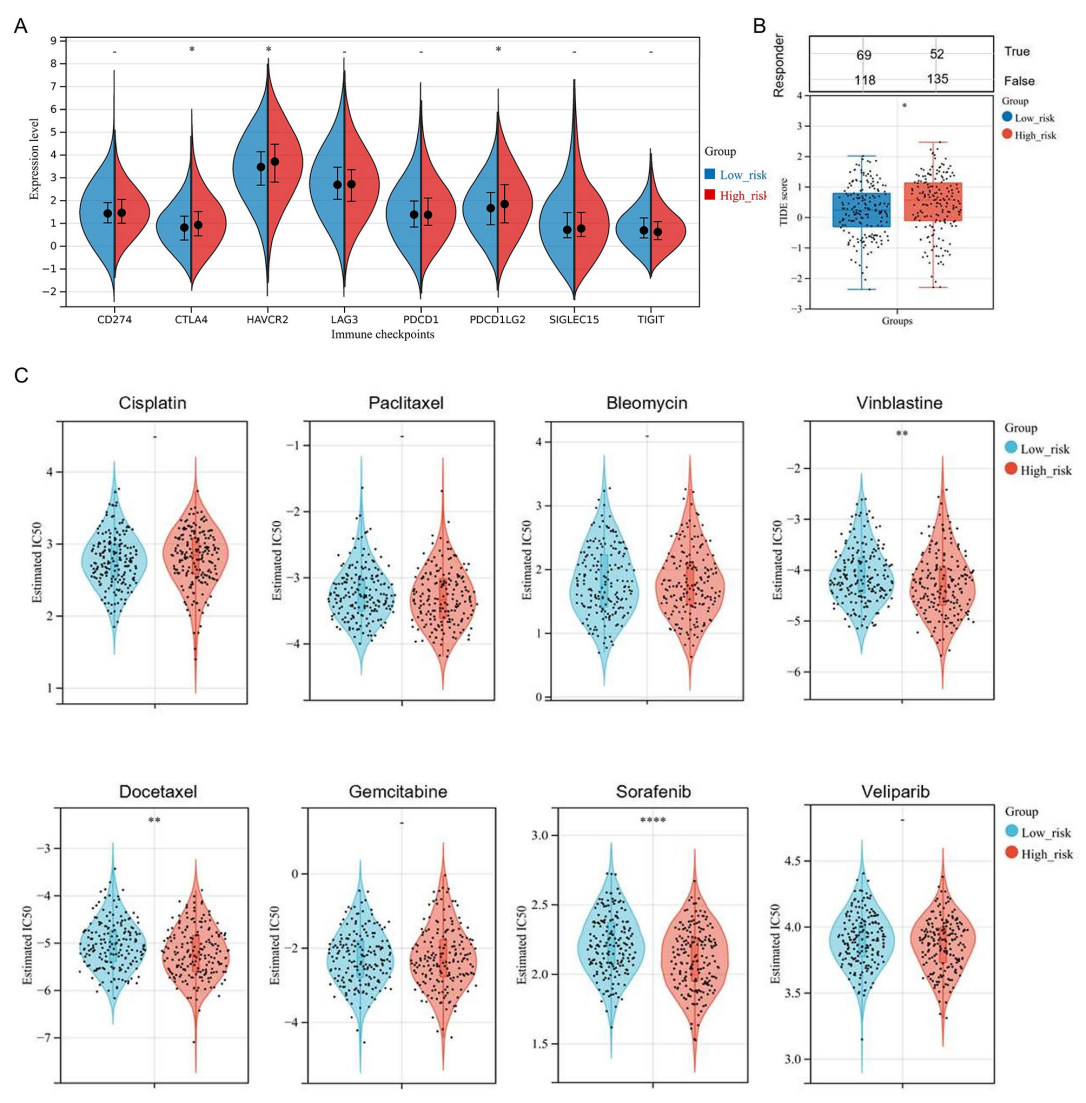
Estimation of the sensitivity to immunotherapy and chemotherapy among OV patients. (**A**) The boxplots for the distribution of 8 typical immune checkpoints gene expression (including CD274, CTLA4, HAVCR2, LAG3, PDCD1, PDCD1LG2, SIGLEC15, and TIGIT) between the two groups classified by the 6-gene signature. (**B**) The immunotherapy response prediction of OV patients, refer to the Tumor Immune Dysfunction and Exclusion(TIDE)score. (**C**) The violin diagrams for the estimated IC50 values distribution for OV patients, in terms of 8 typical chemotherapies, including Bleomycin, Cisplatin, Docetaxel, Gemcitabine, Paclitaxel, Sorafenib, Vinblastine, and Veliparib. The chemotherapy sensitivity analysis was conducted based on the Genomics of Drug Sensitivity in Cancer (GDSC) database. *P-value < 0.05; **P-value < 0.01; ****P-value < 0.0001

In addition, we assessed the association between the gene signature and immune checkpoint molecules expression, which implied that CTLA4, PDCD1LG2, and HAVCR2 were significantly up-regulated among the high-risk group (Fig. 7A, P-value < 0.05). Accordingly, high-risk OV patients were more likely to benefit from the immunotherapies focused on these 3 immune checkpoints. Furthermore, we also predicted patient response to immune checkpoint blockade (ICB) through the Tumor Immune Dysfunction and Exclusion (TIDE) algorithm. Figure 7B showed that high-risk OV patients had higher TIDE scores, which indicated poorer efficacy and shorter survival after ICB therapy (p-value < 0.05).

In order to evaluate chemotherapy sensitivity between two risk groups, we assessed the half-maximal inhibitory concentration (IC50) of 8 typical OV chemotherapy agents through the Genomics of Drug Sensitivity in Cancer (GDSC) dataset. The results implied that the estimated IC50 values of Vinblastine, Docetaxel, and Sorafenib in the high-risk group were significantly lower, compared to those in the low-risk group, indicating that high-risk OV patients were more sensitive to these chemotherapies. However, no significant difference between the two risk groups was found in sensitivity to Cisplatin, Paclitaxel, Bleomycin, Gemcitabine, and Veliparib (p-value ≥ 0.05) (Fig. 7C).

## Discussion

OV is the most fatal gynecological malignant tumors worldwide, mainly due to inefficient biomarkers and high recurrence rates [1, 5]. Therefore, identifying a promising prognostic signature is of great urgency to improve OV survival. Latterly, pyroptosis, a newly-discovered inflammatory form of cell death caused by certain inflammasomes, has been demonstrated to play vital roles in the regulation of tumor progression, thus be considered a potential strategy for tumor treatment [8, 33]. As for OV, previous studies indicated that regulation of PRGs, including HOTTIP [34], α-NETA [35], and LncRNA GAS5 [36] in tumor cells could promote pyroptosis by inducing inflammasome formation, in order to inhibit OV progression, which could be used as a potential target for tumor therapy [7, 37]. Therefore, in this study, we aimed to identify a pyroptosis-related signature and evaluated prognostic potential, tumor immune microenvironment, and sensitivity to treatments related to ferroptosis patterns.

Recently, few current studies focused on pyroptosis, especially on its mechanism in OC. Zhou and colleagues constructed and validated a pyroptosis-related 8-gene signature (including CD44, EPB41L3, FCN1, IRF4, ISG20, LYN, SLC31A2, and VSIG4), which could be used to predict OV prognosis [38]. However, the study only included 25 PRGs for signature identification, which could limit the accuracy and integrality of the research. Another research from Ye and colleagues defined another prognostic signature, which consisted of 8 PRGs including AIM2, CASP3, CASP6, ELANE, GSDMA, PLCG1, and PJVK, though with limited ROC-AUC for 1-year, 2-year, and 3-year OS prediction of 0.628, 0.662, and 0.607, respectively [11]. Up till now, none of the pyroptosis-related prognostic signatures have been standardized and applied to OV clinical practice yet, which might be caused by the limited prognosis value. Accordingly, in our study, we aimed to identify a satisfactory pyroptosis-related signature from 106 potential PRGs obtained from the Genecards database. Through integrative analysis, we distinguished a 6-gene signature (CITED2, EXOC6B, MIA2, NRAS, SETBP1, and TRPV4), which had a promising prognostic value among both training cohorts (TCGA-OV, p-value < 0.0001) and validation cohort (ICGC-OV, p-value = 0.0002). To the best of our knowledge, this is the first study identifying the 6-gene pyroptosis-related OV signature with satisfactory prognostic value, in order to guide clinical decision-making for OV patients.

Among the 6 identified PRGs, only NRAS and SETBP1 have definite functions reported in OV progression. Dariush and colleagues demonstrated that NRAS, an oncogenic driver in serous ovarian carcinomas, could co-expressed with EIF1AX, which promoted clonogenicity and proliferation in OV [24, 25]. SETBP1 was an oncoprotein that directly binds to SET, which could protect it from proteasome degradation [39]. As for OV, Qiao and colleague reported that SETBP1 could maintain the Cancer Stem Cell (CSC)-like phenotype of tumor cells via the SET/PP2A axis [23]. Previous studies identified EXOC6B as a gene involved in the Notch signaling pathway, a key pathway in tumor progression, though haven’t been validated in OV yet [27, 28]. In breast cancer, researchers indicated that CITED2, as a transcriptional coactivator, could modulate the metastatic ability of tumor cells through the regulation on IKKα [29]. Kurihara etc., claimed that MIA2 could regulate the infiltration of lymphocytes via a variety of integrins and subtypes of mitogen-activated protein kinase in oral squamous cell carcinoma [26]. As for TRPV4, researchers found that TRPV4 could promote breast cancer metastasis by regulating cell extravasation, stiffness, and actin cortex [22]. Interestingly, in our study, the Hazard ratios of CITED2 and EXOC6B were greater than 1 in Fig. 2C, and the higher expression of CITED2 and EXOC6B, the poorer survival in Fig. 2F. The results indicated that CITED2 and EXOC6B were risk factors related to OV progression. However, CITED2 and EXOC6B were highly expressed in normal group in Fig. 2E, which demonstrated that CITED2 and EXOC6B were negatively related to oncogenesis. The underlying mechanism for the opposite role of CITED2 and EXOC6B in OV progression and oncogenesis needs further validation and investigation.

Nowadays, owing to the increasing breakthroughs in immune checkpoint inhibitors, the crosstalk between immune environment and tumor has gained increasing attention [40]. Current studies have reported that tumor cells could release signals that recruited anti-tumor immune cells through the pyroptosis process, while the immune cells could also induce pyroptosis in tumor cells, thus causing a positive feedback loop [41, 42]. For instance, Wang and colleagues concluded that pyroptosis of less than 15% of tumor cells was sufficient to eliminate the entire mammary tumor graft, partly due to anti-tumor immunity. In tumors that underwent pyroptosis, the number of CD4 + T cells, CD8 + T cells, NK cells, and M1 macrophages largely increased, while the number of M2 macrophages, monocytes, and neutrophils decreased [43]. Another study by Zhang and colleagues reported that CD8 + T cells and NK cells could evoke pyroptosis of tumor cells independent of caspases through the GSDME-GZMB axis, which is induced by interferon-γ (IFNγ) [44]. Nevertheless, the correlations between immune cell infiltration and pyroptosis patterns in OV remains to be further explored.

Accordingly, we evaluated the landscape of immune infiltration in OV. According to the CIBERSORT analysis, 2 out of the 22 immune cells, including activated DCs and M1 macrophages, were up-regulated in the low-risk group compared to the high-risk group. Lee et al. concluded that activated DCs were essential for T cell recruitment into the tissue, the initiation of T cell responses, and maintenance of effector memory T cells [45]. In this regard, activated DCs played an essential role in the immune responses in the process of OV progression [45, 46]. Most previous studies have reported anti-tumor effects of M1 macrophages, which was consistent with our findings [47]. Surprisingly, Untack Cho and colleagues indicated that M1 macrophages could promote OV metastasis by activating the NF-κB signaling pathway [48]. Interestingly, we also found that Follicular Helper T cells and M2 Macrophage had the most substantial negative relationship, while CD8 + T cells and Macrophage M1 had the strongest positive relationship. However, these findings need validation and exploration for the underlying mechanism in the future study.

Nowadays, regardless of the recent advances in immunotherapy and chemotherapy, clinical treatments for OV face bottlenecks, with a high recurrence rate of approximately 80%, [5, 49]. Emerging evidence demonstrated that pyroptosis, a programmed cell death (PCD) process mediated by gasdermin (GSDM), was a new bridge to tumor immunity, which could influence sensitivity to immunotherapy and chemotherapy [50, 51]. Accordingly, we tried to explore the relationship between pyroptosis patterns and sensitivity to immunotherapy and chemotherapy based on the 6-gene signature. According to the evaluation through the GDSC dataset, high-risk patients were more sensitive to chemotherapy, including Vinblastine, Docetaxel, and Sorafenib. Besides, our results revealed that high-risk patients were more likely to benefit from the immunotherapies based on immune checkpoint molecules, including CTLA4, PDCD1LG2, and HAVCR2. However, OV patients with high risk-score had higher TIDE scores, which indicated poorer efficacy and shorter survival after ICB therapy. Previous researches showed that some PD-L1-positive patients could be insensitive to PD-L1/PD-1 immunotherapy in clinical practice of OV therapy [52]. Hence, our findings might hint that the underlying mechanism of immune checkpoint inhibitors in OV could be more complicated than directly targeting the related immune checkpoints.

However, there remained several limitations of the study. Firstly, the underlying mechanism of the 6 identified PGRs, especially CITED2, EXOC6B, MIA2, and TRPV4, in OV progression and tumor immune microenvironment remained largely unknown, which needs stepwise investigation. Moreover, the pyroptosis-related signature should be further validated in more populations, in order to apply to clinical practice and improve OV survival in the future.

## Conclusion

In brief, our study identified and validated a pyroptosis-related 6-gene signature (consist of CITED2, EXOC6B, MIA2, NRAS, SETBP1, and TRPV4), as a promising prediction tool for treatment response and prognosis in OV. Comprehensive analysis revealed that the pyroptosis-related signature was related to m6A modification and several critical signal pathways in cancer, though the underlying mechanisms remained largely unclear. As for the tumor immune microenvironment, the immune analysis identified significant correlations between immune cell infiltration and pyroptosis patterns, thereby hinted individual treatment of immunotherapy and chemotherapy in specific OV patients. Our findings provided a vital basis for future research focus on the relationship between pyroptosis patterns and tumor immune microenvironment, for the sake of assisting decision-making for OV patients, in the realm of precision medicine.

### Electronic supplementary material

Below is the link to the electronic supplementary material.

**Supplementary Material 1 Table 1** The clinicopathological features of ovarian cancer (OV) patients.

**Supplementary Material 2 Table 2** Overview of the six differentially expressed pyroptosis-related.

**Supplementary Material 3 Figure 1** The Principal Component Analysis (PCA) dimensionality reduction on samples from the TCGA-OV and ovarian normal tissue from GTEx based on their expression of the pyroptosis-related gene signature.

**Supplementary Material 4 Figure 2** The clinical features of OV patients, stratified by the pyroptosis-associated 6-gene signature.
