# Supplementary material for: A Novel pyroptosis-related signature for predicting prognosis and evaluating tumor immune microenvironment in ovarian cancer
Source: J Ovarian Res. 2023 Sep 20;16:196. doi: 10.1186/s13048-023-01275-2 (PMC10512632; doi:10.1186/s13048-023-01275-2)
Supplement: Supplementary file 1 — Supplementary Material 1 Table 1 The clinicopathological features of ovarian cancer (OV) patients. [file 13048_2023_1275_MOESM1_ESM.docx]

Supplement table 1: The clinicopathological features of ovarian cancer (OV) patients from the TCGA-OV cohort and ICGC-OV cohort.

| Features | TCGA-OV cohort (n=376) | ICGC-OV cohort (n=111) | p-value |
| --- | --- | --- | --- |
| Age |  |  | 0.841 |
| <60 years | 206 (54.79%) | 62 (55.86%) |  |
| ≥60 years | 170 (45.21%) | 49 (44.14%) |  |
| FIGO Stage |  |  | 0.026 |
| I-II | 22 (5.85%) | 0 (0.00%) |  |
| III | 296 (78.72%) | 96 (86.49%) |  |
| IV | 58 (15.43%) | 15 (13.51%) |  |
| Grade |  |  | 0.933 |
| I-II | 53 (14.09%) | 16 (14.41%) |  |
| III | 323 (85.90%) | 95 (85.59%) |  |
| Follow-up (months) | 41.76±31.78 | 41.63±31.16 | 0.594 |

Abbreviation: TCGA, The Cancer Genome Atlas database; ICGC, International Cancer Genome Consortium database; FIGO, International Federation of Gynecology and Obstetrics.
