# Supplementary material for: A Novel pyroptosis-related signature for predicting prognosis and evaluating tumor immune microenvironment in ovarian cancer
Source: J Ovarian Res. 2023 Sep 20;16:196. doi: 10.1186/s13048-023-01275-2 (PMC10512632; doi:10.1186/s13048-023-01275-2)
Supplement: Supplementary file 2 — Supplementary Material 2 Table 2 Overview of the six differentially expressed pyroptosis-related. [file 13048_2023_1275_MOESM2_ESM.docx]

**Supplement table 2. Overview of the six differentially expressed pyroptosis-related genes (DE-PRGs) with prognosis value in ovarian cancer (OV).** [22-29]

| Gene symbol | Gene name | Function in OV | Reference |
| --- | --- | --- | --- |
| TRPV4 | Transient Receptor Potential Cation Channel Subfamily V Member 4 | Unknown in OV. TRPV4 could promote breast cancer metastasis by regulating cell stiffness, extravasation, and actin cortex | [22] |
| SETBP1 | SET Binding Protein 1 | SETBP1, as an oncoprotein directly binds to SET to protect it from proteasome degradation, could maintain the Cancer Stem Cell (CSC)-like phenotype of OV cells through the SET/PP2A axis. | [23] |
| NRAS | Neuroblastoma RAS Viral (V-Ras) Oncogene Homolog | NRAS is an oncogenic driver restricted to serous ovarian carcinomas. Co-expression of mutant NRAS and EIF1AX proteins could promote clonogenic survival and proliferation in OV cells | [24, 25] |
| MIA2 | MIA SH3 Domain ER Export Factor 2 | Unknown in OV. The role of MIA2 in oral squamous cell carcinoma is based on the variety of integrins and subtypes of mitogen-activated protein kinase, which is correlated with infiltration of lymphocytes. | [26] |
| EXOC6B | Exocyst Complex Component 6B | Unknown in OV. Previous studies identified EXOC6B as a gene relevant for intellectual development and electrophysiological stability. Moreover, EXOC6B gene was involved in the exocyst/Notch signaling pathways. | [27, 28] |
| CITED2 | Cbp/P300 Interacting Trans activator With Glu/Asp Rich Carboxy-Terminal Domain 2 | Unknown in OV. CITED2, a transcriptional coactivator, could modulate metastatic ability of breast cancer cells through the effects on IKKα. | [29] |
