# Supplementary material for: A Novel pyroptosis-related signature for predicting prognosis and evaluating tumor immune microenvironment in ovarian cancer
Source: J Ovarian Res. 2023 Sep 20;16:196. doi: 10.1186/s13048-023-01275-2 (PMC10512632; doi:10.1186/s13048-023-01275-2)

**Supplement figure 1：The** **Principal Component Analysis (PCA) dimensionality reduction on samples from the TCGA-OV and ovarian normal tissue from GTEx based on their expression of the pyroptosis-related gene signature.** (A) The boxplot of variances gene distribution among the TCGA-OV and GTEx datasets. The PCA analysis of the pyroptosis-related gene signature between the TCGA-OV and GTEx datasets, which was presented by (B) the 2D plot and (C) the 3D plot.


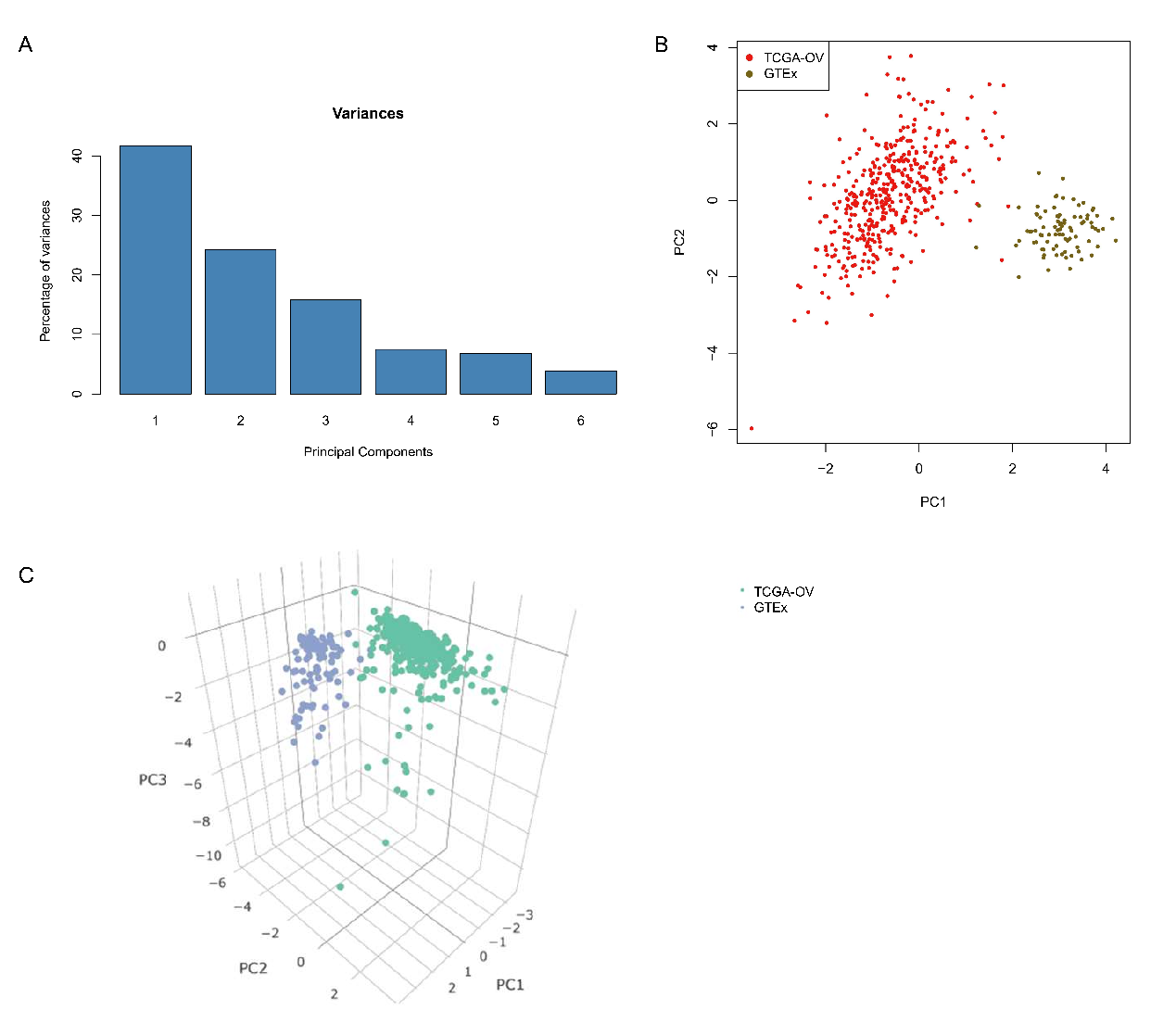

Supplement: Supplementary file 3 — Supplementary Material 3 Figure 1 The Principal Component Analysis (PCA) dimensionality reduction on samples from the TCGA-OV and ovarian normal tissue from GTEx based on their expression of the pyroptosis-related gene signature. [file 13048_2023_1275_MOESM3_ESM.docx]
