# Supplementary material for: A Novel pyroptosis-related signature for predicting prognosis and evaluating tumor immune microenvironment in ovarian cancer
Source: J Ovarian Res. 2023 Sep 20;16:196. doi: 10.1186/s13048-023-01275-2 (PMC10512632; doi:10.1186/s13048-023-01275-2)
Supplement: Supplementary file 4 — Supplementary Material 4 Figure 2 The clinical features of OV patients, stratified by the pyroptosis-associated 6-gene signature. [file 13048_2023_1275_MOESM4_ESM.docx]

**Supplement figure 2. The clinical features of OV patients, stratified by the pyroptosis-associated 6-gene signature.** (A-D) The stacked bar diagram for the distribution of various clinical characteristics, including age, race, pathological grade, and clinical FIGO stage, between low-risk and high-risk groups. (E) The Sankey plot of clinical characteristics, including age, pathological grade, clinical FIGO stage, and the 6-gene signature. Each row represented a different feature, while each line indicated the distribution of sample, refer to different features. *P-value < 0.05; NS, no significance.

**
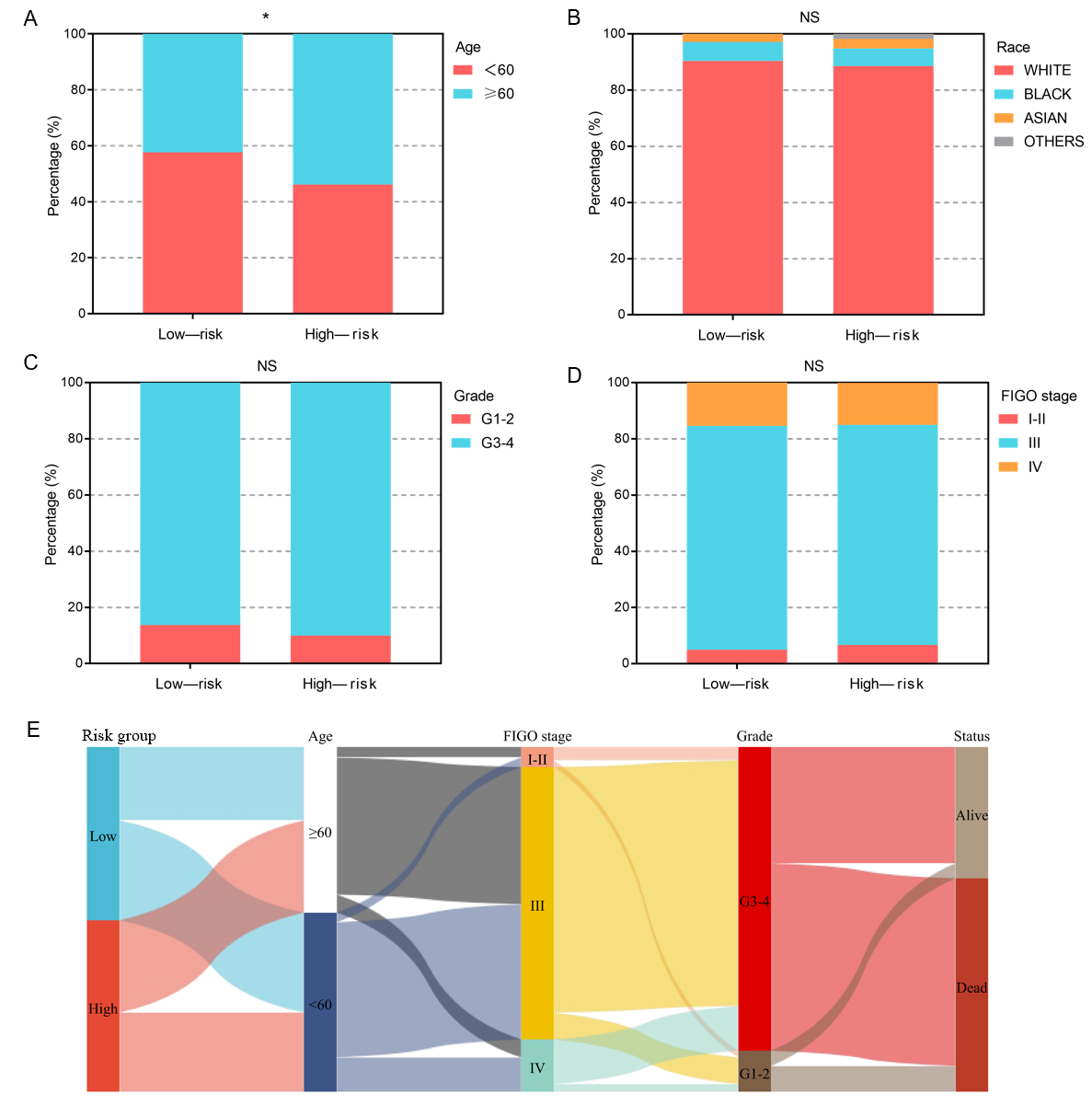
**
